# Phenotypic detection of multidrug resistance and metallo-β-lactamase production among clinically relevant non-fermenters at a medical centre in India

**DOI:** 10.6026/973206300212080

**Published:** 2025-07-31

**Authors:** Somya Sinha, Shireen Shailja, Namrata Kumari, Shailesh Kumar

**Affiliations:** 1 Department of Microbiology, SNMIH, Saharsa, Bihar, India; 2 Department of Microbiology, Shyamlal Chandrashekhar Medical College, Khagaria, Bihar, India; 3 Department of Microbiology, IGIMS, Patna, Bihar, India

**Keywords:** Non-fermenting gram-negative bacilli (NFGNB), multidrug resistance (MDR), metallo-beta-lactamase (MBL), tertiary care hospital, nosocomial infections, colistin

## Abstract

Non-fermenting gram-negative bacilli (NFGNB) have become a new vision of serious and clinically important nosocomial infection, with a growing multidrug resistance (MDR), as well as the production of metallo-beta-lactamase (MBL), which makes their intervention in tertiary care centres difficult. This 18-month prospective research carried out in Bihar scrutinized 342 NFGNB isolates, whereby the most dominant was *Pseudomonas aeruginosa*. MDR was observed in 72.8 percent of isolates and it was significantly higher in people in the ICU (p < 0.001), whereas MBL was seen in 41.2 percent of strains. The highest susceptibility was shown by colistin, polymyxin B and tigecycline. Thus, the urgent need for effective antimicrobial stewardship and infection control practices is reported.

## Background:

Non-fermenting gram-negative bacilli (NFGNB) are a heterogeneous group of opportunistic pathogens that have risen as major causes of healthcare-related diseases all around [1]. Owing to the close association with high morbidity and mortality rates, these organisms, identified by their failure to digest glucose and natural resistance to most antimicrobial agents, have become relevant in the clinical microbiology field [2]. The major clinically significant non-fermenting Gram-negative bacilli (NFGNB) include *Pseudomonas aeruginosa*, *Acinetobacter baumannii*, *Stenotrophomonas maltophilia* and the *Burkholderia cepacia* complex, which are known to cause severe infection even in immunocompromised persons and other individuals with previous comorbid conditions [3]. Multidrug resistance (MDR) against non-fermenting Gram-negative bacteria (NFGNB) has become a considerable global health challenge, as non-fermenting Gram-negative bacteria exhibit resistance to almost all commonly used antibiotics, such as antipseudomonal penicillins, cephalosporins, aminoglycosides, fluoroquinolones and carbapenems. Recent studies have made alarming reports of MDR-NFGNB infections among individuals with a range of 60 to 90 percent frequency as reported in various hospital settings [4]. This is worsened by the fact that production of metallo-beta-lactamases (MBLs), the production of which renders it resistant to carbapenems, which in most instances are considered to be the last resort drugs used to treat severe infections of gram-negative bacteria [5]. Metallo-beta-lactamases are also zinc-dependent enzymes and break down all beta-lactam antibiotics, excluding aztreonam and the encoding genes are frequently transferred to mobile genetic elements and facilitate rapid transfer to bacterial communities [5]. The emergence of NFGNB producing MBL has been associated with high mortality rates, lengthy stays in hospitals and healthcare spending [6]. There are various phenotypic methods of identifying the MBL production that have been put in place, including using a combination disk test using imipenem-EDTA, which is one of the most common screening tests used in any clinical laboratory [7]. The epidemiology of NFGNB infection has a significant disparity in a number of geographical locations and health setups. Various studies have pointed out that there is a high prevalence of MDR-NFGNB in India and tertiary care institutions have shown prevalence rates of 40-80 percent [8]. Bihar is one of the most populous states of India that faces unique challenges in dealing with antibiotic resistance because of a poorly developed healthcare system. The alarming changes in patterns of resistance to common antibiotics against gram-negative bacteria have been documented, especially in tertiary care facilities in the state of Bihar [9]. Although the clinical significance of NFGNB infections continues to mount, little comprehensive information is known about the prevalence, as well as the antibiotic resistance dynamics of these pathogens in the area of Bihar. Furthermore, there is not enough information and evidence of the prevalence of MBL-producing NFGNB in this region, which is necessary in making appropriate empirical treatment guidelines and prevention control measures against the infections [10]. Therefore, it is of interest to ascertain the prevalence of multidrug resistance and metallo-beta-lactamase production among clinically significant non-fermenting gram-negative bacilli isolated from diverse clinical specimens at a tertiary care center in Bihar and to assess their antimicrobial susceptibility patterns to inform rational antibiotic therapy.

## Materials and Methods:

The field work of this prospective observational study was conducted in the Department of Microbiology, IGIMS, Patna, Bihar, during the span of 18 months, spanning October 2018 to March 2020. Authors obtained ethical permission from a confirmed Institutional Ethics Committee and informed consent on the side of participants or their guardians. Every sequential and non-redundant bacteriological specimen that was submitted consecutively to the culture of the bacteria, *i.e.*, blood, urine, sputum, pus, wound swabs, cerebrospinal fluid and pleural fluid, was worked on. They only considered clinically relevant non-fermenting gram-negative bacilli (NFGNB) isolates, excluding the repeat isolates of the same patient within 30 days. Antimicrobial Susceptibility Testing: There are several clinical guidelines on how bacteria are identified and the testing of antimicrobial susceptibility. Preliminary isolation was performed by use of blood agar and MacConkey agar, to which they were then further incubated at 37°C. Normal biochemical test was first used to identify NFGNB, but conclusive identification was done by using the VITEK 2 Compact system (bioMerieux, France). The Kirby-Bauer disk diffusion method was used as the antimicrobial susceptibility testing method against drugs, which include piperacillin-tazobactam, ceftazidime, imipenem, meropenem, amikacin, colistin, polymyxin B and tigecycline, according to the CLSI standards. Quality control was performed on *E. coli* ATCC 25922 and *P. aeruginosa* ATCC 27853 strains.

## Definitions and MBL identification:

Multidrug resistance (MDR) is known as non-susceptibility to at least one agent in three or more antimicrobial categories. Extensively drug-resistant (XDR) isolates showed non-susceptibility to more than two or fewer types of medication. Two phenotypic methods were used to determine the production of metallo-beta-lactamase (MBL) in the carbapenem-resistant isolates: a combined imipenem-EDTA disk test (a zone difference of at least 7 mm was indicative of positive results) and the Modified Hodge Test, which showed carbapenemase production by producing greater growth in *E. coli* that surrounded the test organism.

## Statistical assessment and data management:

Systematic recording of clinical and demographic data was done. Parameters were provided through statistical means using SPSS 26.0. Categorical variables were calculated in frequencies and percentages, but continuous ones in means and standard deviations. Group comparisons were done using the Chi-square test and student t-test and the p-value of less than 0.05 was considered statistically significant.

## Results:

A total of 342 clinically significant non-fermenting gram-negative bacilli (NFGNB) were determined in a sample of 7,428 clinical specimens with an isolation rate of 4.6%. The average age of patients was 48.72 18, 2 years, with the majority being males (57.90) percent. In patients, most isolates were obtained in the inpatients (78.1%). *Pseudomonas aeruginosa* (58.2%) was identified as the most common isolated species, followed by *Acinetobacter baumannii* (28.4%), *Stenotrophomonas maltophilia* (7.9%) and *Burkholderia cepacia* (5.5). The most frequently used source of isolates was respiratory specimens (37.4%), pus/wound (26.0%), blood (19.6%) and urine (17.0%), in that order (Table 1 - see PDF). Antimicrobial susceptibility determined that the resistance rates were very high in the majority of the antibiotic classes. Piperacillin-tazobactam (78.4 percent), ceftazidime (81.3 percent), cefepime (76.9 percent), imipenem (69.3 percent) and meropenem (71.1 percent) resistance were found. The resistance towards amikacin and gentamicin was 68.7 and 79.5 percent, by aminoglycosides. Fluoroquinolone resistance was also elevated, 84.2 percent to ciprofloxacin and 79.8 percent to levofloxacin. The greatest activity was maintained by colistin (89.5%), polymyxin B (85.1%) and tigecycline (76.3). *A. baumannii* had the highest quantity of species that were resistant to carbapenems and aminoglycosides. MDR was 72.8% and XDR phenotype was observed in 31.3% of isolates; the highest XDR was detected in *A. baumannii* with 52.6 % (Table 2 - see PDF). The proportion of the totals of NFGNB isolates that produced MBLs was 41.2 percent; 56.0 percent of the carbapenem-resistant isolates. The positivity of MBL was the highest in *A. baumannii* (67.5%) and below in *P. aeruginosa* (38.7%). Modification of the Hodge test recognized more carbapenemase activity in 18.7 percent of MBL-negative, carbapenem-resistant isolates. The two tests together were also able to check carbapenemase production in 74.6 percent of carbapenem-resistant isolates. The duration of hospital stay and the death rates in patients with MDR or MBL-positive infection were significantly longer (p < 0.001). Multivariate logistic regression indicated ICU admission, previous antibiotic exposure, mechanical ventilation and length of hospital stays >7 days as independent risk factors of MDR-NFGNB infections (Table 3 - see PDF and Table 4 - see PDF).

## Discussion:

This paper presents detailed information about the distribution and antimicrobial resistance of NFGNB of a tertiary care facility in Bihar state, which is alarming as there is a high prevalence of multidrug resistance and production of metallo-beta-lactamases. The general prevalence of MDR overall of 72.8% as we have observed in our study, is similar to the recent findings of other tertiary care centers in India, where the prevalence of MDR in NFGNB has been reported at a level of 60-85 per cent [11]. This prevalence rate is indicative of the difficult therapeutic situation that clinicians experience when trying to treat NFGNB infections in resource-limited areas. The high rate of *P. aeruginosa* (58.2%) and *A. baumannii* (28.4%) among the NFGNB isolates reflects the trend at the global epidemiological level, where these pathogens have been reported to be at the top of the list in the causes of healthcare-associated infections [12]. Isolation rate of 37.4 percent and 26.0 percent in respiratory specimens and wound samples, respectively, was consistent with the opportunistic nature of the pathogens, which frequently resulted in ventilator-associated pneumonia and postsurgical wound infections among hospitalized patients [13]. The pattern of antimicrobial susceptibility showed widespread resistance to the widely employed antibiotics, with over 75 percent of the most common beta-lactam antibiotics and fluoroquinolones presenting resistance. The results may be compared to recent ones in the tertiary care hospitals in India, where these patterns of resistance have also been reported [14]. It is especially worrying that the carbapenem resistance was also high (imipenem 69.3%, meropenem 71.1%) since these agents are usually deemed as the last resort in the treatment of serious gram-negative infections [15]. It is an important discovery that the MBL production can be identified in 41.2 percent of the NFGNB isolates since such enzymes are resistant to all but two antibiotics (aztreonam) and they are characterized by limited therapeutic alternatives [16]. A higher percentage of *A. baumannii* produces MBL (67.5%) than *P. aeruginosa* (38.7%) agrees with the earlier results by the Indian hospitals and *A. baumannii* emerged to be a key reservoir of MBL genes [17]. The clinical significance of these resistant pathogens was a matter of long hospitalizations and increased mortality of patients with the presence of MBL-producing organisms. The fact that colistin and polymyxin B turned out to be the most effective agents (89.5 per cent and 85.1 per cent of susceptibilities, respectively) is an indication of the therapeutic reality of the contemporary methods of MDR-NFGNB infections therapy. Nevertheless, the fact that colistin resistance has already been observed even at such minimum levels is alarming, as there are very few alternatives to such therapy [18]. The comparatively higher activity of tigecycline (76.3 percent sensitive) offers another alternative agent, mainly against *A. baumannii* infection [19]. The high correlation of MDR-NFGNB infection in ICU admission (84.3% vs 65.7 %, p < 0.001) highlights the aspect of intensive care settings as niches of such resistant organisms. This observation is in line with surveillance data at the global level that indicate greater prevalence rates of antimicrobial resistance in ICUs because of several factors that include greater selection pressure of the antimicrobials, invasive procedures and cross-acquisition between patients [20]. Variants of the previous exposure to antibiotics and MDR highlight the significance of antimicrobial stewardship initiatives in mitigating the kind of pressure that promotes the growth of resistant organisms [21]. Hospital stay is much longer in MDR-NFGNB infections (18.6 +/- 7.2 days vs 12.4 +/- 4.8 days), which is in turn a critical indication of impact on the cost and resources. Other studies related to this established similar results with MDR-NFGNB infections being linked to longer stay, higher expenditure on treatment and greater healthcare burden [22]. Antimicrobial resistance has an economic effect not only in terms of direct medical expenses but also in lost productivity and societal costs [23]. The fact that 74.6 percent of carbapenem-resistant isolates showed carbapenemase production when screened using a combination of phenotypic tests indicates that the screening tools do produce valuable results in resource-limited areas where molecular diagnostic techniques are not easily accessible [24]. The Modified Hodge Test detected more carbapenemase-producing isolates than those that were detected by the MBL screening test, which indicates the need for various test methods during surveillance [25]. The strong sides of our study are the large volume of research, the prospective study design and extensive antimicrobial susceptibility testing of standardized methods. Diverse phenotypic procedures of MBL screening present strong values of MBL decreasing mechanisms' generalization. Some limitations are, however, warranted. The research was implemented in only one center and thus, the outcomes may not apply to other health facilities. The absence of molecular characterization of resistance genes does not allow carrying out the fine analysis of certain variants of MBL and their correlation with the epidemiology of infections [26]. The clinical significance of the results of this research study is significant to clinical practice and the politics of health. The prevalence of MDR and MBL-producing NFGNB is very high and therefore, this requires instalment of strong infection control measures, such as improved surveillance, contact precautions and environmental decontamination methods. It is of concern that the emergence of resistance can also be prevented through the development of antimicrobial stewardship programs involving the rational use of antibiotics, specifically carbapenems [27]. A process of regular surveillance of the patterns of antimicrobial resistance trends should be implemented in order to influence empirical therapy recommendations and epidemiological trends. Research with emphasis on molecular analysis of resistant mechanisms, new treatments and combination therapy for the management of MDR-NFGNB infections should be undertaken in the future. The discovery of rapid and sensitive diagnostic tools that are located at the POC to identify the mechanisms of resistance will significantly contribute to the clinical decision-making process and the outcomes associated with increased patient recovery rates [28].

## Conclusion:

The current analysis shows the prevalence of multidrug resistance (72.8%) and of MBL producers (41.2%) of NFGNB isolates in a tertiary care center in the state of Bihar. The widespread antibiotic resistance, particularly to carbapenem, highlights the necessity of urgently implementing active antimicrobial stewardship and strong infection control. Quick detection and surveillance are important to control the infection effectively.
